# Resting and Postexercise Heart Rate Detection From Fingertip and Facial Photoplethysmography Using a Smartphone Camera: A Validation Study

**DOI:** 10.2196/mhealth.7275

**Published:** 2017-03-13

**Authors:** Bryan P Yan, Christy KY Chan, Christien KH Li, Olivia TL To, William HS Lai, Gary Tse, Yukkee C Poh, Ming-Zher Poh

**Affiliations:** ^1^ Division of Cardiology Department of Medicine and Therapeutics The Chinese University of Hong Kong and Prince of Wales Hospital Hong Kong China (Hong Kong); ^2^ Faculty of Medicine Newcastle University Newcastle upon Tyne United Kingdom; ^3^ Li Ka Shing Institute of Health Sciences Faculty of Medicine The Chinese University of Hong Kong Hong Kong China (Hong Kong); ^4^ Cardiio Inc Cambridge, MA United States

**Keywords:** heart rate, mobile apps, photoplethysmography, smartphone, mobile phone

## Abstract

**Background:**

Modern smartphones allow measurement of heart rate (HR) by detecting pulsatile photoplethysmographic (PPG) signals with built-in cameras from the fingertips or the face, without physical contact, by extracting subtle beat-to-beat variations of skin color.

**Objective:**

The objective of our study was to evaluate the accuracy of HR measurements at rest and after exercise using a smartphone-based PPG detection app.

**Methods:**

A total of 40 healthy participants (20 men; mean age 24.7, SD 5.2 years; von Luschan skin color range 14-27) underwent treadmill exercise using the Bruce protocol. We recorded simultaneous PPG signals for each participant by having them (1) facing the front camera and (2) placing their index fingertip over an iPhone’s back camera. We analyzed the PPG signals from the Cardiio-Heart Rate Monitor + 7 Minute Workout (Cardiio) smartphone app for HR measurements compared with a continuous 12-lead electrocardiogram (ECG) as the reference. Recordings of 20 seconds’ duration each were acquired at rest, and immediately after moderate- (50%-70% maximum HR) and vigorous- (70%-85% maximum HR) intensity exercise, and repeated successively until return to resting HR. We used Bland-Altman plots to examine agreement between ECG and PPG-estimated HR. The accuracy criterion was root mean square error (RMSE) ≤5 beats/min or ≤10%, whichever was greater, according to the American National Standards Institute/Association for the Advancement of Medical Instrumentation EC-13 standard.

**Results:**

We analyzed a total of 631 fingertip and 626 facial PPG measurements. Fingertip PPG-estimated HRs were strongly correlated with resting ECG HR (*r*=.997, RMSE=1.03 beats/min or 1.40%), postmoderate-intensity exercise (*r*=.994, RMSE=2.15 beats/min or 2.53%), and postvigorous-intensity exercise HR (*r*=.995, RMSE=2.01 beats/min or 1.93%). The correlation of facial PPG-estimated HR was stronger with resting ECG HR (*r*=.997, RMSE=1.02 beats/min or 1.44%) than with postmoderate-intensity exercise (*r*=.982, RMSE=3.68 beats/min or 4.11%) or with postvigorous-intensity exercise (*r*=.980, RMSE=3.84 beats/min or 3.73%). Bland-Altman plots showed better agreement between ECG and fingertip PPG-estimated HR than between ECG and facial PPG-estimated HR.

**Conclusions:**

We found that HR detection by the Cardiio smartphone app was accurate at rest and after moderate- and vigorous-intensity exercise in a healthy young adult sample. Contact-free facial PPG detection is more convenient but is less accurate than finger PPG due to body motion after exercise.

## Introduction

There are over 100,000 health-related apps in the health and fitness category in the Google Play Store and the iTunes App Store designed for mobile devices (ie, smartphones or tablets) [1,2]. The number of health-related apps is increasing by 25% each year [3]. Mobile device usage has constantly been on the rise over the last few years [4], with an estimated 6.9 billion subscriptions globally [5]. In the United States, 64% of adults own at least one smartphone [6], and 62% of smartphone owners have used their phone to obtain health information. In Europe, 50% of citizens own a smartphone [7]. In the Asia Pacific region [8], the number of smartphone users is estimated to reach 1.2 billion in 2017. With technological advances and the increasing trend of mobile device usage, mHealth is becoming popular and is seen as an opportunity to promote health and fitness, provide health maintenance, or enhance lifestyle management [9-11]. Compared with home health devices and computers, personal mobile devices with health-related apps installed are more convenient, portable, and accepted [12,13]. Older adults are more likely to own a smartphone than a computer [11]. An estimated 19 million people use mobile health devices worldwide [14].

Over the last few years, multiple smartphone devices and apps were developed to facilitate heart rate (HR) monitoring. Monitoring of HR during exercise can be used to assess fitness level and intensity of exercise. It is also useful for patients who are taking medications that affect HR to guide disease management. Recently, several smartphone apps capable of measuring HR and detecting arrhythmia have also been reported [15,16]. Modern smartphones allow measurement of HR by detecting pulsatile photoplethysmographic (PPG) signals with built-in cameras from the fingertips or the face without physical contact by extracting subtle beat-to-beat variations of skin color that is similar to HR fluctuations [17,18]. The PPG signal is typically recorded by placing a finger over the camera lens, which measures color changes due to fingertip blood volume changes [19]. Facial PPG recording using the smartphone camera is a novel method of detecting pulsatile PPG signal without physical contact [20,21].

HR estimation using the built-in smartphone cameras may provide readily accessible, inexpensive, and user-friendly means to measure HR without additional hardware such as wrist bands or watches. However, validation of HR measurements detected from smartphone-based PPG apps is limited [22-24]. This study aimed to evaluate the accuracy of HR measurements at rest and after exercise using the Cardiio-Heart Rate Monitor + 7 Minute Workout (Cardiio) smartphone-based PPG detection app compared with a continuous electrocardiogram (ECG) as the reference.

## Methods

### Participants and Recruitment

We recruited 40 healthy participants between 18 and 40 years old with no current medical conditions and who were not taking regular medications. Demographics characteristics were collected. Body height and weight were measured under standard anthropometry procedures, and body mass index was calculated as weight in kilograms divided by height in square meters. Blood pressure was measured using an automatic blood pressure monitor (Tango M2, SunTech Medical, Inc., NC, USA) before and after testing procedures. The participants were evaluated for facial skin color by using the von Luschan skin color chart (range 1-36) [25]. This study was approved by the Joint Chinese University of Hong Kong – New Territories East Cluster Clinical Research Ethics Committee (CREC Ref. No. 2016.550).

### Study Setup

We set up 2 iPhones (iPhone 6S; Apple, Inc, Cupertino, CA, USA) and a 12-lead ECG treadmill (GE Series 2000, GE Medical Systems Information Technologies Inc, Milwaukee, WI, USA) for HR measurements. Backdrop and background light intensity was standardized during signal acquisition and was captured in the unit of lux. The Cardiio (Cardiio Inc, Cambridge, MA, USA) smartphone app was installed in the 2 iPhones for facial and fingertip PPG detection. HR measurements were taken by continuous 12-lead ECG, and facial and fingertip PPG detection simultaneously. Each participant was given at least a 5-minute rest interval before testing began. We took 3 measurements of resting HR and averaged them for analysis before exercise. Participants were then tested on a motorized treadmill using the Bruce test protocol from stage 1 to stage 4 (2.7, 4.0, 5.5, and 6.8 km/h) [26]. Each participant underwent 2 sequential tests on the treadmill to achieve (1) moderate-intensity exercise, defined as 50% to 70% maximum HR, and (2) vigorous-intensity exercise, defined as 70% to 85% maximum HR. Maximum HR was calculated as 220 beats/min minus the participant’s age [27]. HR recordings were acquired immediately when moderate- and vigorous-intensity exercise was achieved based on HR from the continuous ECG and repeated successively until return to resting HR.

### Heart Rate Measurements

#### 12-Lead Electrocardiogram

Continuous 12-lead ECG was our reference standard of HR measurement. We compared HRs detected by the Cardiio smartphone app with simultaneous 12-lead ECG recordings.

#### Facial PPG Detection

**Figure 1:**
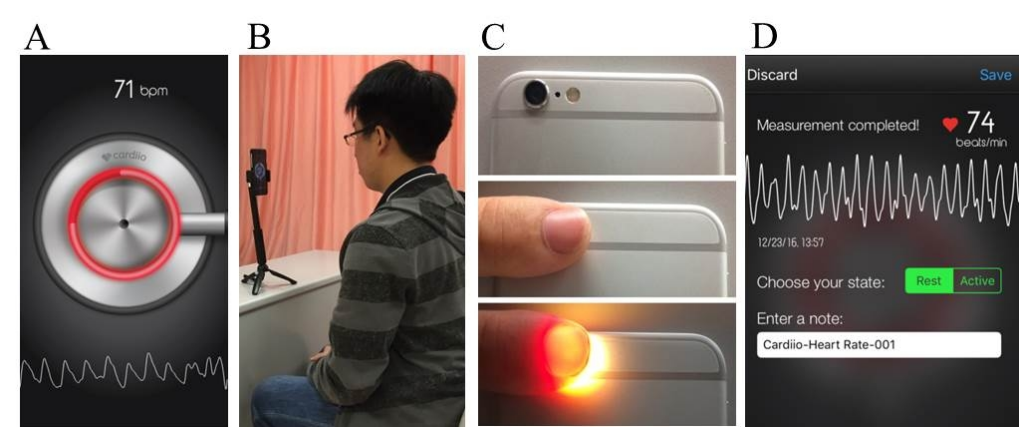
(A) Cardiio smartphone app. (B) Setup to acquire photoplethysmographic (PPG) signals from the participant's face. (C) Obtaining PPG signals from the fingertip. (D) Example of a report produced by the Cardiio smartphone app.

We asked participants to sit in front of an iPhone placed upright on a desk approximately 30 cm away. Once the Cardiio smartphone app was activated, a large circle appeared on the screen, and each participant needed to position the image of his or her entire face within the circle so that it was captured by the front camera (Figure 1). Participants were instructed to hold still for 20 seconds during each measurement. The continuous pulsatile PPG signal from the face detected by the camera was displayed on the bottom of the iPhone screen. An estimated HR measurement analyzed by the Cardiio smartphone app was displayed as a result. Participants were asked to keep their movements to a minimum and not to speak during measurement.

#### Fingertip PPG Detection

Fingertip PPG signals were recorded for each participant by placing their index fingertip over the iPhone’s back camera for 20 seconds. Once the finger was placed in contact and illuminated by the adjacent LED flash, a continuous pulsatile PPG signal from the fingertip was detected by the camera and displayed on the bottom of the iPhone screen (Figure 1). It was analyzed by the Cardiio smartphone app for an estimated HR measurement and displayed as a result. Participants were asked to keep their movements to a minimum and not to speak during measurement.

### Statistical Analysis

We present numerical results as mean (SD) or median (interquartile range, IQR). Pearson correlation (*r*) and coefficient of determination were applied to determine the nature of the relationship between the HR measurements made by the Cardiio smartphone app (face and fingertip PPG detection) and by the standard 12-lead ECG. We performed the paired Student *t* test and Wilcoxon signed rank test to determine the difference between the means and medians measured by both devices. Root mean square error (RMSE) was performed to evaluate the spread of errors between predicted and observed values. Bland-Altman plots were used to examine agreement between ECG and PPG-estimated HR. All statistical analyses were performed using IBM SPSS statistical software (IBM SPSS Statistics for Windows, version 22.0; IBM Corporation). All analyses were 2-tailed, and *P* values of <.05 were considered statistically significant.

We recorded a total of 665 fingertip and 665 facial PPG measurements. Boxplots were used to determine extreme outliers for any observation outside the upper and lower fences that were 3 times the IQR. Potential outliers were checked for accuracy before exclusion. Failed measurements (n=4 facial measures) and extreme outliers were possibly caused by the monitor losing fingertip skin contact or misalignment of the face. We excluded 69 outliers for PPG measurements representing unrealistic HR values (n=34 fingertip measures and n=35 facial measures), resulting in 631 fingertip and 626 facial PPG measurements for analysis.

## Results

### Characteristics of Participants

Table 1 summarizes participants’ characteristics. Of the 40 healthy participants, 50% (n=20) were men, and the mean (SD) age was 24.7 (5.2) years. The median von Luschan skin color among participants was 23 (IQR 19-25); the lightest skin color was 14 and the darkest was 27. Male participants had a higher body mass index, resting systolic blood pressure (SBP), and resting diastolic blood pressure (DBP) than the female participants. There were no significant differences in postexercise SBP and DBP between the sexes. The median backdrop and background light intensity during signal acquisition was 199 lux (IQR 127-249).

**Table 1:** Baseline characteristics of the study participants.

Variables	Male (n=20)	Female (n=20)	All participants (N=40)	*P* value
Age in years, mean (SD)	23.9 (4.6)	25.5 (5.8)	24.7 (5.2)	.34
von Luschan skin color, median (IQR^a^)	23.5 (22-24)	19 (18-25.75)	23 (19-25)	.19
Height in cm, mean (SD)	172.5 (5.8)	160.1 (5.9)	166.3 (8.5)	<.001
Weight in kg, mean (SD)	69.0 (10.2)	51.8 (5.4)	60.4 (11.9)	<.001
Body mass index in kg/m^2^, mean (SD)	23.1 (2.4)	20.2 (1.7)	21.6 (2.5)	<.001
Resting SBP^b^ in mmHg, mean (SD)	132.6 (15.0)	117.0 (17.2)	124.8 (17.8)	<.001
Resting DBP^c^ in mmHg, mean (SD)	78.7 (11.1)	72.8 (9.0)	75.7 (10.5)	.01
Postexercise SBP in mmHg, mean (SD)	146.3 (20)	135.1 (29.1)	140.7 (25.4)	.05
Postexercise DBP in mmHg, mean (SD)	73.4 (12.1)	74.9 (19.9)	74.2 (16.3)	.68

^a^IQR: interquartile range.

^b^SPB: systolic blood pressure.

^c^DBP: diastolic blood pressure.

### Fingertip PPG-Estimated Heart Rate

We analyzed 80 averaged resting HR values from 234 PPG measurements, and overall 397 postmoderate- and postvigorous-intensity exercise HR values.

For resting HR, the Cardiio app captured 6% (5/80) of HR values ≥100 beats/min, and the mean (SD) HR was 73.41 (12.60) beats/min (range 52.67-112 beats/min) (Table 2). The mean (SD) difference in HR between fingertip PPG measurements and the reference ECG was –0.05 (1.03) beats/min (*P*=.69, paired *t* test; Table 3).

**Table 2:** Comparison of heart rates (HRs) measured by electrocardiogram (ECG), and fingertip and facial photoplethysmography (PPG).

Activity		Device	n	HR≥100 beats/min, n (%)	HR (beats/min)		
					Mean (SD)	Minimum	Maximum
**Fingertip PPG detection**							
	**Resting**						
		ECG	80	5 (6)	73.46 (12.74)	52.67	114.00
		Cardiio	80	5 (6)	73.41 (12.60)	52.67	112.00
	**Postmoderate-intensity exercise**						
		ECG	177	51 (28.8)	90.33 (19.85)	51	142
		Cardiio	177	50 (28.2)	89.97 (19.95)	50	145
	**Postvigorous-intensity exercise**						
		ECG	220	132 (60.0)	105.30 (20.07)	58	157
		Cardiio	220	133 (60.5)	105.12 (19.91)	57	157
**Facial PPG detection**							
	**Resting**						
		ECG	80	5 (6)	73.46 (12.74)	52.67	114.00
		Cardiio	80	5 (6)	73.17 (12.64)	51.50	112.67
	**Postmoderate-intensity exercise**						
		ECG	188	55 (29.3)	90.44 (19.33)	51	142
		Cardiio	188	56 (29.8)	89.35 (19.47)	52	138
	**Postvigorous-intensity exercise**						
		ECG	201	114 (56.7)	103.46 (19.14)	58	157
		Cardiio	201	110 (54.7)	102.53 (19.16)	59	158

**Table 3:** Accuracy of measuring heart rate (HR) using Cardiio smartphone app compared with reference electrocardiogram.

Statistic		Resting HR	Postmoderate-intensity exercise HR	Postvigorous intensity-exercise HR
**Fingertip PPG^a^detection**				
	Coefficient of determination	.993	.988	.990
	RMSE^b^ (beats/min)	1.03	2.15	2.01
	RMSE (%)	1.40	2.53	1.93
	Wilcoxon signed rank test, *P* value	.53	.09	.60
	Paired Student *t* test, *P* value	.69	.03	.19
	Mean (SD) difference (beats/min)	–0.05 (1.03)	–0.36 (2.14)	–0.18 (2.02)
	Median difference (beats/min)	0.500	0	0
**Facial PPG detection**				
	Coefficient of determination	.994	.965	.960
	RMSE (beats/min)	1.02	3.68	3.84
	RMSE (%)	1.44	4.11	3.73
	Wilcoxon signed rank test, *P* value	.02	<.001	.08
	Paired Student *t* test, *P* value	.01	<.001	.001
	Mean (SD) difference (beats/min)	–0.29 (1.03)	–1.09 (3.67)	–0.93 (3.84)
	Median difference (beats/min)	–1.166	–2.000	–1.000

^a^PPG: photoplethysmography.

^b^RMSE: root mean square error.

The mean (SD) HR of postmoderate-intensity exercise was 89.97 (19.95) beats/min (range 50-145 beats/min) with 28.2% (50/177) of HR values ≥100 beats/min captured by the Cardiio app. For postvigorous-intensity exercise, the mean (SD) HR was 105.12 (19.91) beats/min (range 57-157 beats/min) with 60.5% (133/220) of HR values ≥100 beats/min captured (Table 2). The mean (SD) differences in HR between fingertip PPG measurements and the reference ECG were –0.36 (2.14) beats/min (*P*<.05) for postmoderate-intensity exercise and –0.18 (2.02) beats/min (*P*=.19) for postvigorous-intensity exercise (Table 3).

**Figure 2:**
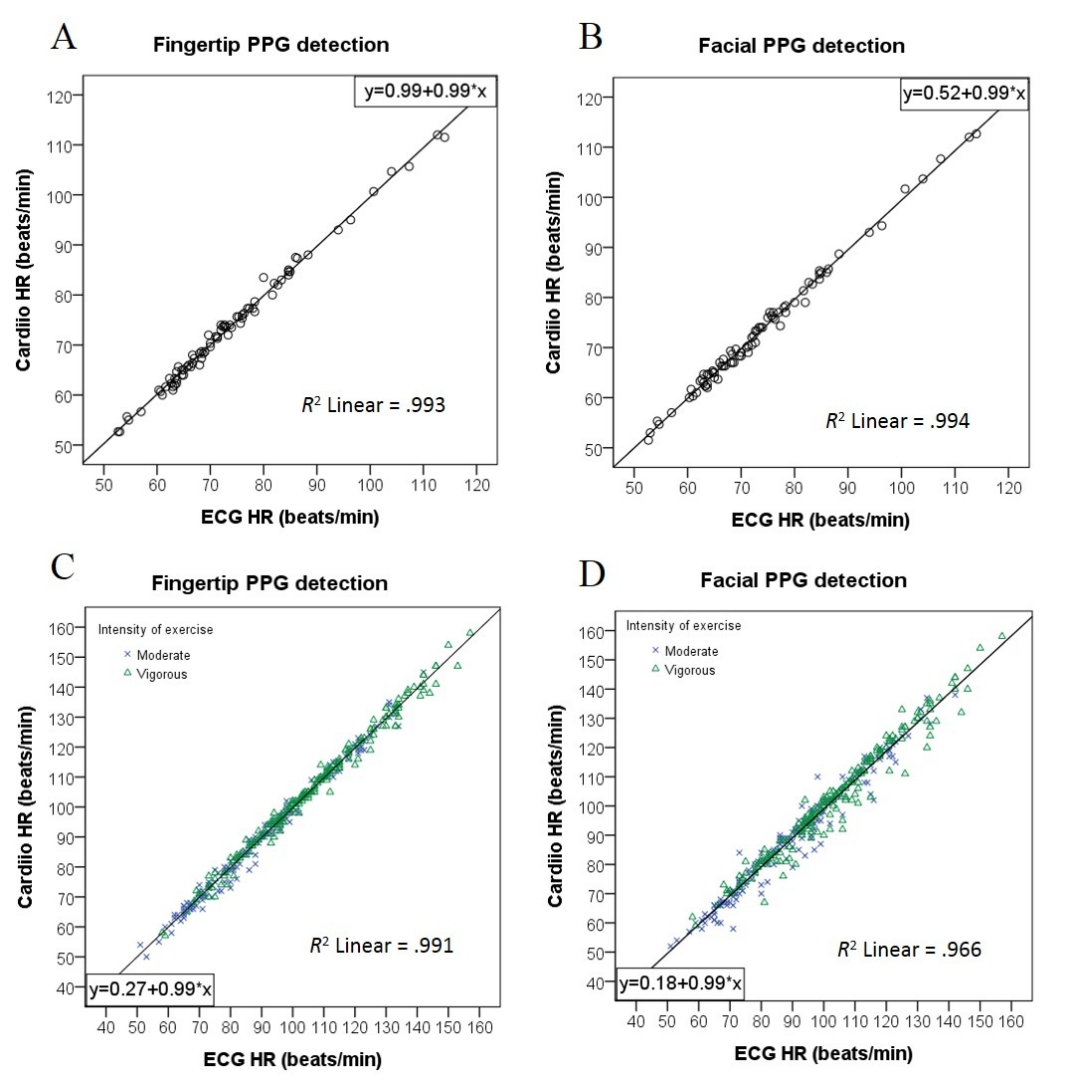
Scatter plots comparing measurements of heart rate (HR) estimated from the Cardiio smartphone phone app photoplethysmographic (PPG) signals and from a reference electrocardiogram (ECG). *P*<.001 for all correlations. (A) Resting estimated HR from fingertip PPG signals. (B) Resting estimated HR from facial PPG signals. (C) Postexercise HR from fingertip PPG signals. (D) Postexercise HR from facial PPG signals.

Table 4 shows correlation coefficients of HR between the Cardiio smartphone app and the reference ECG. Fingertip PPG-estimated HRs were strongly correlated with ECG HR at rest (*r*=.997, *P*<.001; RMSE=1.03 beats/min or 1.40%), postmoderate-intensity exercise (*r*=.994, *P*<.001; RMSE=2.15 beats/min or 2.53%), and postvigorous-intensity exercise (*r*=.995, *P*<.001; RMSE=2.01 beats/min or 1.93%), as Figure 2 illustrates.

**Table 4:** Correlation test (*r*)^a^ of heart rate between reference electrocardiogram (ECG) and Cardiio smartphone app.

Activity	ECG	Fingertip PPG^b^ detection	Facial PPG detection
Resting	1	.997	.997
Postmoderate-intensity exercise	1	.994	.982
Postvigorous-intensity exercise	1	.995	.980
Postexercise overall	1	.995	.983

^a^*P*<.001 for all correlations (correlation is significant at the .01 level, 2-tailed).

^b^PPG: photoplethysmography.

**Figure 3:**
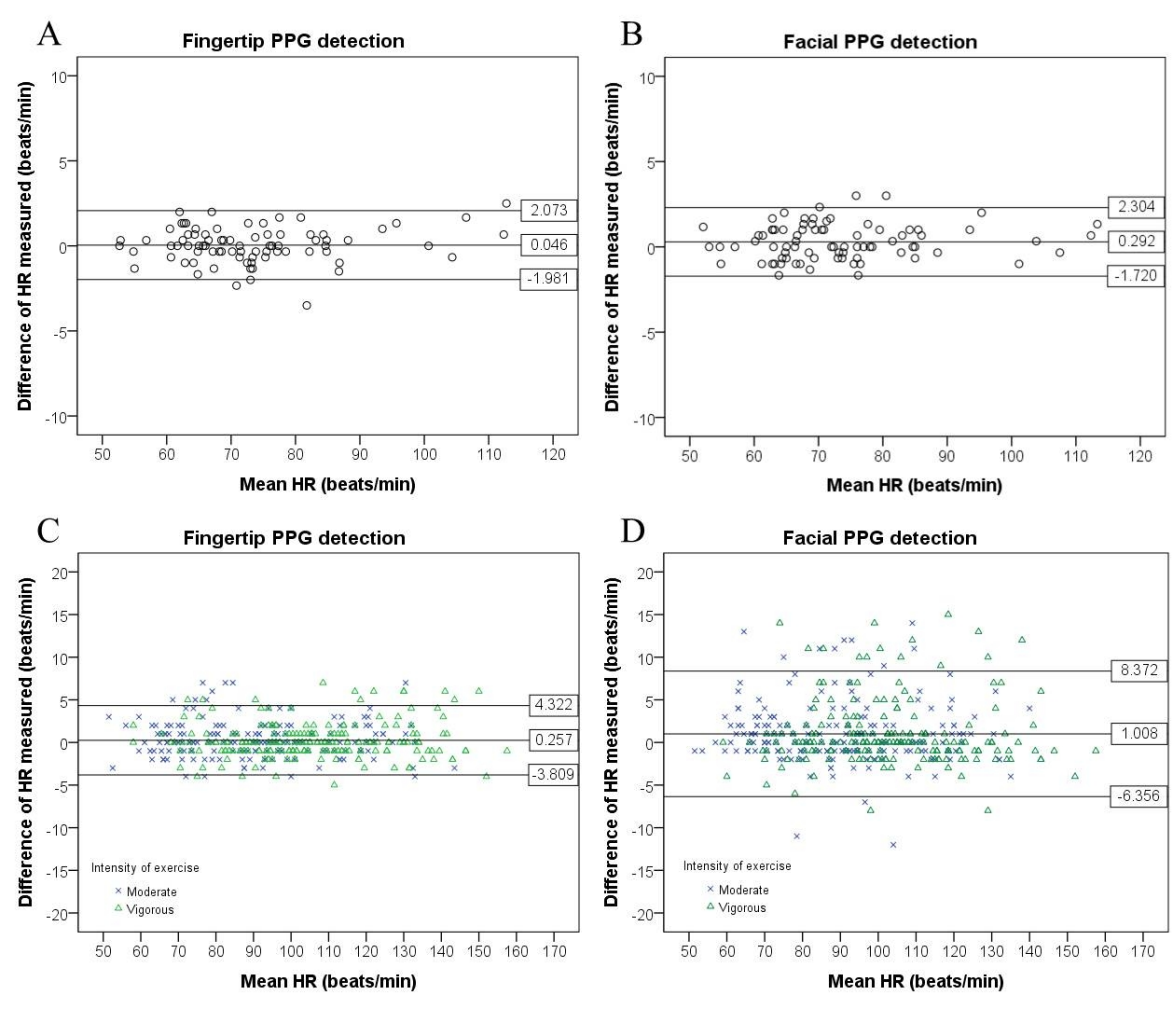
Bland-Altman plots of limits of agreement in resting heart rate (HR) estimated from the Cardiio smartphone app and a reference electrocardiogram (ECG). (A) Resting estimated HR from fingertip PPG signals. (B) Resting estimated HR from facial PPG signals. (C) Postexercise HR from fingertip PPG signals. (D) Postexercise HR from facial PPG signals.

Figure 3 presents the Bland-Altman plots with 95% limits of agreement. The Cardiio smartphone app had 95% of resting HR measurements fall within –1.98 and +2.07 beats/min, while postexercise HR measurements were within –3.81 and +4.32 beats/min.

### Facial PPG-Estimated Heart Rate

We analyzed 80 averaged resting HR values from 237 facial PPG measurements and a total of 389 postexercise HR values.

The mean (SD) HRs were 73.17 (12.64) beats/min (range 51.50-112.67 beats/min) at rest, 89.35 (19.47) beats/min (range 52-138 beats/min) postmoderate-intensity exercise, and 102.53 (19.16) beats/min (range 59-158 beats/min) postvigorous-intensity exercise (Table 2). Facial PPG detection determined 6% (5/80) of resting, 29.8% (56/188) of postmoderate-intensity exercise, and 54.7% (110/201) of vigorous-intensity exercise HR values ≥100 beats/min (Table 2).

The mean (SD) difference in HR between facial PPG detection and the reference ECG at rest was –0.29 (1.03) beats/min (*P*<.05), while postmoderate- and postvigorous-intensity exercise HR differences were –1.09 (3.67) beats/min (*P*<.05) and –0.93 (3.84) beats/min (*P*<.05), respectively (paired *t* test comparing fingertip PPG detection with reference ECG, Table 3).

The HR values from facial PPG measurements and the reference ECG were positively correlated (Table 4). The correlation of facial PPG-estimated HR with ECG HR (*r*=.997, *P*<.001; RMSE=1.02 beats/min or 1.44%) was strong at rest. Facial PPG-estimated HR was also strongly correlated with ECG HR after exercise under moderate intensity (*r*=.982, *P*<.001; RMSE=3.68 beats/min or 4.11%) and vigorous intensity (*r*=.980, *P*<.001; RMSE=3.84 beats/min or 3.73%) (Figure 2).

The Cardiio smartphone app had 95% of resting HR measurements fall within –1.72 and +2.30 beats/min, while postexercise HR measurements were wider and within –6.36 and +8.37 beats/min, as illustrated in the Bland-Altman plots (Figure 3).

## Discussion

### Principal Findings

According to the literature, HR monitors are considered accurate and regarded as excellent when *r* ≥.93 and RMSE<6.8% [28,29]. The American National Standards Institute/Association for the Advancement of Medical Instrumentation EC-13 standard states that the accuracy requirements for HR monitors are RMSE≤5 beats/min or ≤10%, whichever is greater [30]. Our results showed that the Cardiio smartphone app using both fingertip and facial PPG detection can be regarded as excellent and considered accurate in measuring HR at rest, and after moderate- to vigorous-intensity exercise.

PPG estimation of resting HR from both the fingertip and the face demonstrated very high accuracy. The RMSE were less than 1.5% (RMSE=1.40% from fingertip and RMSE=1.44% from facial PPG measurements). The errors between PPG-estimated resting HR and ECG HR were <1%, as illustrated by the coefficients of determination (Figure 2).

In particular, the results of fingertip PPG measurements were consistent and accurate in the different physical activity levels tested in this study. Fingertip PPG-estimated HRs were strongly correlated with both resting and postexercise ECG HR (all *r* ≥.99; Table 4). HR was underestimated with mean differences of <0.5 beats/min or median differences of 0 beats/min at rest and after exercise compared with the reference ECG (Table 3).

For facial PPG measurements, the accuracy of HR estimation was better at rest and diminished with exercise. The coefficient of determination of facial PPG-estimated HR was stronger with resting ECG HR (*R*^2^>.99) than postexercise ECG HR (*R*^2^≥.96) (Table 3). For postexercise HR detection, facial PPG underestimated HR values with mean differences of approximately 1 beat/min or median differences of 1-2 beats/min when compared with ECG HR (Table 3). Possible explanations are blushing and excessive facial motion due to heavy breathing after exercise leading to misalignment of the face with the camera.

Overall, the Bland-Altman plots showed better agreement between ECG and fingertip-estimated HR than between ECG and facial PPG-estimated HR, and that 95% limits of agreement were wider for facial than for fingertip PPG estimation (Figure 3). The results demonstrated the ability of a PPG-based smartphone app to provide meaningful and accurate readings at rest and after exercise.

### Limitations

Limitations of our study include that (1) this was a convenience sample of healthy young adults, which may limit generalizability; and (2) the method of HR detection from fingertip and facial PPG using iPhone’s front and back cameras, which may not apply to other smartphone cameras. We recommend further evaluation of the accuracy of taking HR measurements in a real-world environment under nonstandardized background lighting and in expanded exercise settings. We suggest conducting further studies including a larger number of participants with an extended age range and more skin tone colors to increase generalizability. This study did not include patients with arrhythmia or other heart-related problems, which is an area for future investigation. Future work could also study user feedback relating to health-sensing mobile apps.

### Conclusions

Smartphone use is becoming ubiquitous. People are increasingly relying on smartphones and health-related apps for health care purposes. Smartphone apps must be validated for their accuracy, reliability, and effectiveness in providing health care benefits. Capturing a PPG signal with the built-in smartphone cameras may provide a readily accessible and inexpensive means to measure HR. Our results have demonstrated that HR detection by the Cardiio smartphone app is accurate at rest and after moderate- and vigorous-intensity exercise in a healthy young adult sample. Both fingertip and facial PPG have shown high accuracy in measuring resting HR with reference to an ECG. Although touchless facial PPG detection is more convenient, fingertip PPG is more accurate for HR detection after exercise.

## Abbreviations

DBP: diastolic blood pressure
ECG: electrocardiogram
HR: heart rate
IQR: interquartile range
PPG: photoplethysmography
RMSE: root mean square error
SBP: systolic blood pressure
