# Figure Correction: Resting and Postexercise Heart Rate Detection From Fingertip and Facial Photoplethysmography Using a Smartphone Camera: A Validation Study

**DOI:** 10.2196/11616

**Published:** 2019-01-03

**Authors:** Bryan P Yan, Christy KY Chan, Christien KH Li, Olivia TL To, William HS Lai, Gary Tse, Yukkee C Poh, Ming-Zher Poh

**Affiliations:** 1 Division of Cardiology Department of Medicine and Therapeutics The Chinese University of Hong Kong and Prince of Wales Hospital Hong Kong China (Hong Kong); 2 Faculty of Medicine Newcastle University Newcastle upon Tyne United Kingdom; 3 Li Ka Shing Institute of Health Sciences Faculty of Medicine The Chinese University of Hong Kong Hong Kong China (Hong Kong); 4 Cardiio Inc Cambridge, MA United States

In “Resting and Postexercise Heart Rate Detection From Fingertip and Facial Photoplethysmography Using a Smartphone Camera: A Validation Study” (JMIR Mhealth Uhealth 2017;5(3):e33), there was an error in Figure 2. The scatter plot of Figure 2C (Postexercise HR from fingertip PPG signals) was a duplicate of  Figure 2D (Postexercise HR from facial PPG signals). The scatter plot of Figure 2C has been updated with a correct value of *R*^2^=0.991. The equation of line (y=0.27+0.99*x) remains unchanged.

The correction will appear in the online version of the paper on the JMIR website on January 3, 2019, together with the publication of this correction notice. Because this was made after submission to PubMed, PubMed Central, and other full-text repositories, the corrected article also has been resubmitted to those repositories.

**Figure 2:**
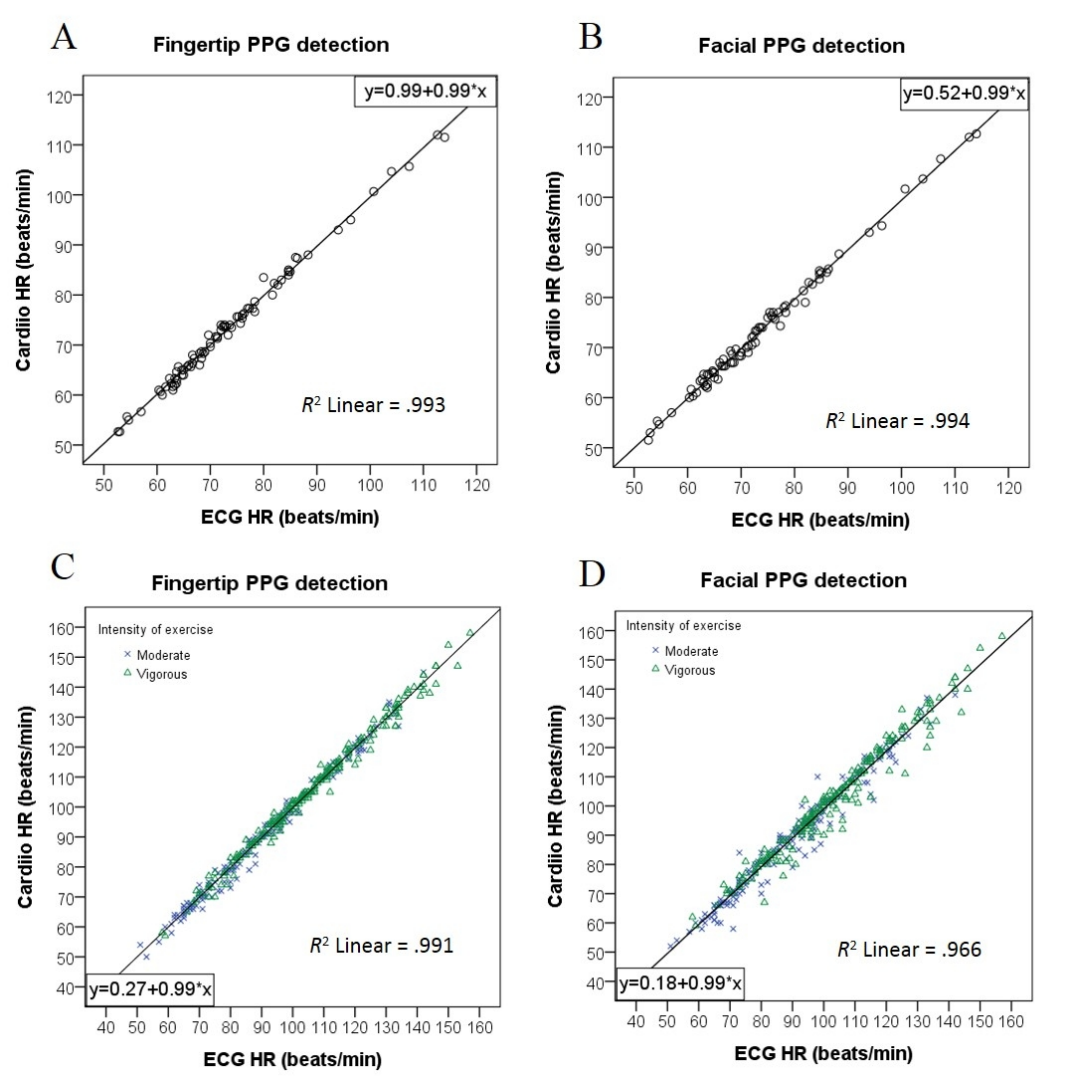
Scatter plots comparing measurements of heart rate (HR) estimated from the Cardiio smartphone phone app photoplethysmographic (PPG) signals and from a reference electrocardiogram (ECG). *P*<.001 for all correlations. (A) Resting estimated HR from fingertip PPG signals. (B) Resting estimated HR from facial PPG signals. (C) Postexercise HR from fingertip PPG signals. (D) Postexercise HR from facial PPG signals.

